# A Comprehensive Prognostic Analysis of POLD1 in Hepatocellular Carcinoma

**DOI:** 10.1186/s12885-022-09284-y

**Published:** 2022-02-21

**Authors:** Hui Tang, Tingting You, Zhao Sun, Chunmei Bai

**Affiliations:** Department of Medical Oncology, Peking Union Medical College Hospital, Chinese Academy of Medical Sciences, Peking Union Medical College, 100730 Beijing, China

**Keywords:** POLD1, Hepatocellular Carcinoma, The Cancer Genome Atlas database, Prognosis, Immunotherapy

## Abstract

**Background:**

DNA polymerase delta 1 catalytic subunit (POLD1) plays a key role in DNA replication and damage repair. A defective DNA proofreading function caused by POLD1 mutation contributes to carcinogenesis, while POLD1 overexpression predicts poor prognosis in cancers. However, the effect of POLD1 in hepatocellular carcinoma (HCC) is not well-understood.

**Methods:**

Expression patterns of POLD1 were evaluated in TCGA and the HPA databases. Kaplan-Meier curves and Cox regression were used to examine the prognostic value of POLD1. The prognostic and predictive value of POLD1 was further validated by another independent cohort from ICGC database. The influences of DNA copy number variation, methylation and miRNA on POLD1 mRNA expression were examined. The correlation between infiltrating immune cells and POLD1 expression was analyzed. GO and KEGG enrichment analyses were performed to detect biological pathways associated with POLD1 expression in HCC.

**Results:**

POLD1 was overexpressed in HCC (*n* = 369) compared with adjacent normal liver (*n* = 50). POLD1 upregulation was significantly correlated with positive serum AFP and advanced TNM stage. Kaplan–Meier and multivariate analyses suggested that POLD1 overexpression predicts poor prognosis in HCC. DNA copy gain, low POLD1 methylation, and miR‑139-3p downregulation were associated with POLD1 overexpression. Besides, POLD1 expression was associated with the infiltration levels of dendritic cell, macrophage, B cell, and CD4 + T cell in HCC. Functional enrichment analysis suggested “DNA replication”, “mismatch repair” and “cell cycle” pathways might be involved in the effect of POLD1 on HCC pathogenesis. Additionally, POLD1 mRNA expression was significantly associated with tumor mutation burden, microsatellite instability, and prognosis in various tumors.

**Conclusions:**

POLD1 may be a potential prognostic marker and promising therapeutic target in HCC.

**Supplementary Information:**

The online version contains supplementary material available at 10.1186/s12885-022-09284-y.

## Background

Hepatocellular carcinoma (HCC) is one of the most common primary tumors worldwide [1]. Unfortunately, the effect of drug treatment on HCC is limited, while the recurrence rate after surgery is about 70% at 5 years [1]. The World Health Organization speculated that more than 1 million patients will die of HCC in 2030 [1]. Therefore, it is urgent to further explore the underlying mechanisms of HCC carcinogenesis and development, which will contribute to the detection of novel promising prognostic and therapeutic targets.

DNA polymerase delta 1 catalytic subunit (POLD1) encodes the 125-kDa catalytic subunit and provides the essential catalytic activity of DNA polymerase delta, which exhibits both DNA polymerase and 3’ to 5’ exodeoxyribonuclease activity, and plays a crucial role in DNA replication, DNA damage repair, cell growth and differentiation [2, 3]. Previous studies suggested that POLD1 was upregulated in HCC and breast cancer, and its overexpression correlated with tumor progression and poor prognosis [4, 5]. Furthermore, POLD1 proofreading (exonuclease) domain mutations were associated with a deficient proofreading repair during DNA replication and an increased incidence of epithelial cancers, especially colorectal and endometrial cancer [6–8]. Recent studies demonstrated that POLD1 proofreading domain mutation can potentially predict desirable outcomes in cancer patients treated with immune-checkpoint inhibitors (ICIs) [9, 10]. However, the effect and underlying mechanisms of POLD1 in HCC are not well-understood.

In the present research, the comprehensive prognostic and predictive value of POLD1 in a well-defined HCC cohort from The Cancer Genome Atlas (TCGA) were first analyzed. The findings were further examined using an independent HCC cohort retrieved from International Cancer Genome Consortium (ICGC) database. The underlying mechanisms of POLD1 in HCC carcinogenesis and development were further analyzed by bioinformatics methods.

## Methods

### Data source and processing

The data (including clinical data, mRNA-seq data, and miRNA-seq data) of 369 primary HCC and 50 adjacent normal liver samples were downloaded from TCGA-LIHC (liver hepatocellular carcinoma) dataset in February 2021. After excluding those without complete Tumor-Node-Metastasis (TNM) stage and follow-up data, 339 HCC patients were enrolled. The validation cohort of 207 primary HCC and 175 adjacent normal liver samples were downloaded from ICGC-LIRI (liver cancer - RIKEN, Japan) dataset in March 2021. Somatic mutation data of TCGA pan-cancer cohort were downloaded from TCGA using UCSC Xena (http://xena.ucsc.edu) [11] in March 2021.

### Gene expression analysis

POLD1 mRNA expression data in several different kinds of tumor tissues and normal controls were retrieved from GEPIA2 (http://gepia2.cancer-pku.cn), a handy tool to explore TCGA and GTEx datasets [12]. Immunohistochemistry (IHC) staining data of POLD1 in HCC and normal liver samples were retrieved from the Human Protein Atlas (HPA, http://www.proteinatlas.org) [13]. In the HPA database, protein expression rank was assessed by the staining intensity (strong/moderate/weak/negative) and fraction of stained cells (> 75%/25–75%/< 25%), including high (strong with > 25%), medium (strong with < 25%, or moderate with > 25%), low (moderate with < 25%, or weak with > 25%), and not detected (weak or negative with < 25%) [14].

The relationship between POLD1 mRNA expression and POLD1 DNA methylation or copy number alterations (CNA) in TCGA-LIHC were determined by cBioPortal for Cancer Genomics (http://www.cbioportal.org) [15] using TCGA-LIHC dataset. Moreover, the association between POLD1 mRNA expression and immune infiltrates in HCC was examined by TIMER2 (http://timer.cistrome.org) [16].

### Functional enrichment analysis

The top 50 positively and negatively POLD1-correlated co-expression genes based on the TCGA-LIHC tumor tissues were retrieved from LinkedOmics (http://www.linkedomics.org) [17]. While the top 100 positively or negatively POLD1-correlated genes based on the TCGA-LIHC tumor and normal liver samples were retrieved from GEPIA2. The protein-protein interaction (PPI) network between POLD1 and other proteins, as well as 50 available experimentally determined POLD1-binding proteins, was retrieved from STRING (http://string-db.org) [18]. Furthermore, the GO (Gene ontology) enrichment analysis was conducted with the “clusterProfiler” package of R software using the combination of 100 POLD1-correlated genes and 50 POLD1-binding proteins. Besides, the KEGG (Kyoto encyclopedia of genes and genomes) enrichment analysis was performed by DAVID (https://david.ncifcrf.gov) [19] using the combination of the above two datasets. The enriched pathways were finally visualized with the “tidyr” and “ggplot2” packages of R software.

### Tumor mutation burden and microsatellite instability in pan-cancer

Tumor mutation burden (TMB) was defined as the total number of nonsynonymous mutations per megabase, and microsatellite instability (MSI) was calculated by the incidence of insertion or deletion that occurred in repeating sequences of genes. TMB scores were calculated with somatic mutation data of TCGA pan-cancer cohort using a Perl script, and adjusted by dividing by the total length of exons. MSI data were derived from previously published research [20]. The results of correlation analysis between POLD1 expression and TMB or MSI were presented as radar plots, generated using the “fmsb” package of R software.

### Statistical analyses

Receiver operating characteristic (ROC) curve was applied to examine the prognostic value of POLD1 expression using the “ROCR” package of R software, and the area under the curve (AUC) was calculated by the “verification” package. The correlation between POLD1 mRNA expression and the clinicopathological variables was evaluated by Pearson’s Chi-square test. POLD1 expression between different clinicopathological groups was evaluated by the “edgeR” package of R software using Quasi-likelihood F-test. Adjusted P value < 0.05 and |log2 FC (fold change)| > 1 were considered to be statistically significant. Propensity-score matching (PSM) was performed to mitigate the influence of confounding factors. The propensity scores were calculated by the “MatchIt” package of R software using multivariable logistic regression based on age, gender, family history, and residual tumor after surgery. We matched propensity scores 1:1 with the nearest neighbor method without replacement by using a 0.02 calipers width. The overall survival (OS) and disease-free interval (DFI) between the low and high POLD1 expression groups were compared by Kaplan–Meier analysis. Cox regression analysis was performed by the “finalfit” package of R software to calculate the hazard ratios (HR) and confidence intervals (CI) of factors associated with OS and DFI in HCC patients. According to the result of multivariate Cox analysis, the nomogram was established by the “rms” package of R software and assessed by the calibration curves and concordance index (C-index). Two-tailed P value < 0.05 was regarded to be statistically significant.

Strawberry Perl (version 5.30.0, http://strawberryperl.com) was applied to extract POLD1 expression data from downloaded datasets. All statistical analyses and visualization were conducted using R software (version 3.6.1, https://www.r-project.org/).

## Result

### Increased POLD1 expression in HCC

**Fig. 1:**
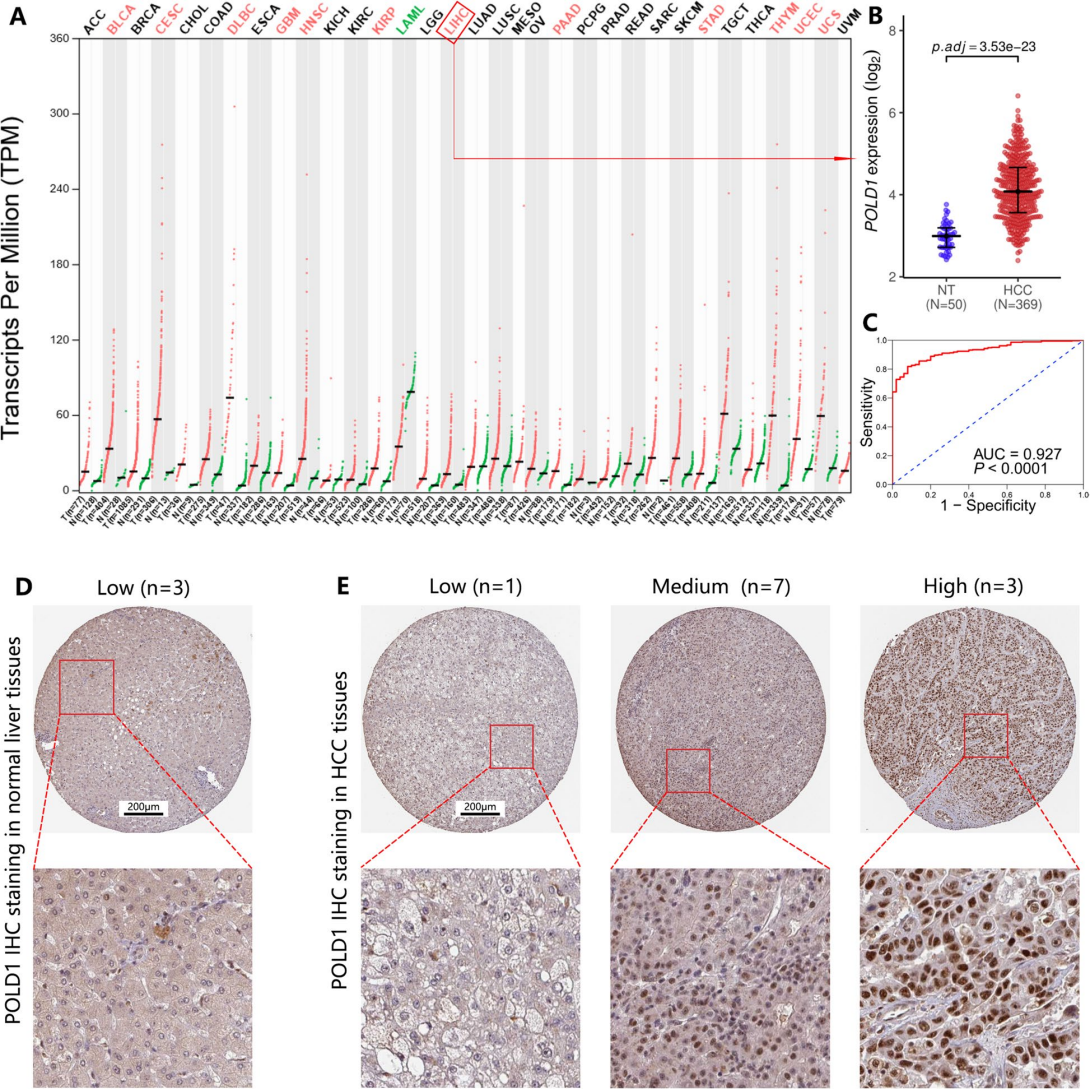
POLD1 is significantly upregulated at the mRNA and protein levels in HCC compared with that in normal liver samples. **A** POLD1 mRNA expression in several tumor and normal controls (data were retrieved from GEPIA2)*. **B** Comparison of POLD1 mRNA expression in HCC (*n* = 369) and in normal liver samples (*n* = 50). **C** Validation of diagnostic value of POLD1 upregulation for HCC using ROC curve. Representative IHC images of POLD1 retrieved from the HPA in normal liver samples **D** and HCC **E**, scale bar 200 μm. *Each dot represents a distinct tumor (red) or normal (green) sample. The cancer names in red font indicates that POLD1 expression was upregulated in the tumor samples compared with the normal samples, the green font indicates that the expression was downregulated, and the black font indicates no significant difference between the tumor and normal samples

POLD1 mRNA expression in several tumors and normal controls was retrieved from GEPIA2 (Fig. 1 A). In TCGA-LIHC dataset, POLD1 expression was significantly upregulated in HCC tissues (*n* = 369) compared with adjacent normal liver samples (*n* = 50) (*P* < 0.0001) (Fig. 1B). The ROC curve (AUC = 0.927, *P* < 0.0001) suggested POLD1 upregulation was related to the diagnosis of HCC (Fig. 1 C). Moreover, the IHC data retrieved from the HPA was used to examine the expression of POLD1 at protein level. While normal liver samples usually displayed low POLD1 staining (3/3), most HCC tissues displayed medium (7/11) or high (3/11) POLD1 staining, which is mainly located in the nucleus (Fig. 1D, E).

### POLD1 overexpression correlated with HCC progression

**Fig. 2:**
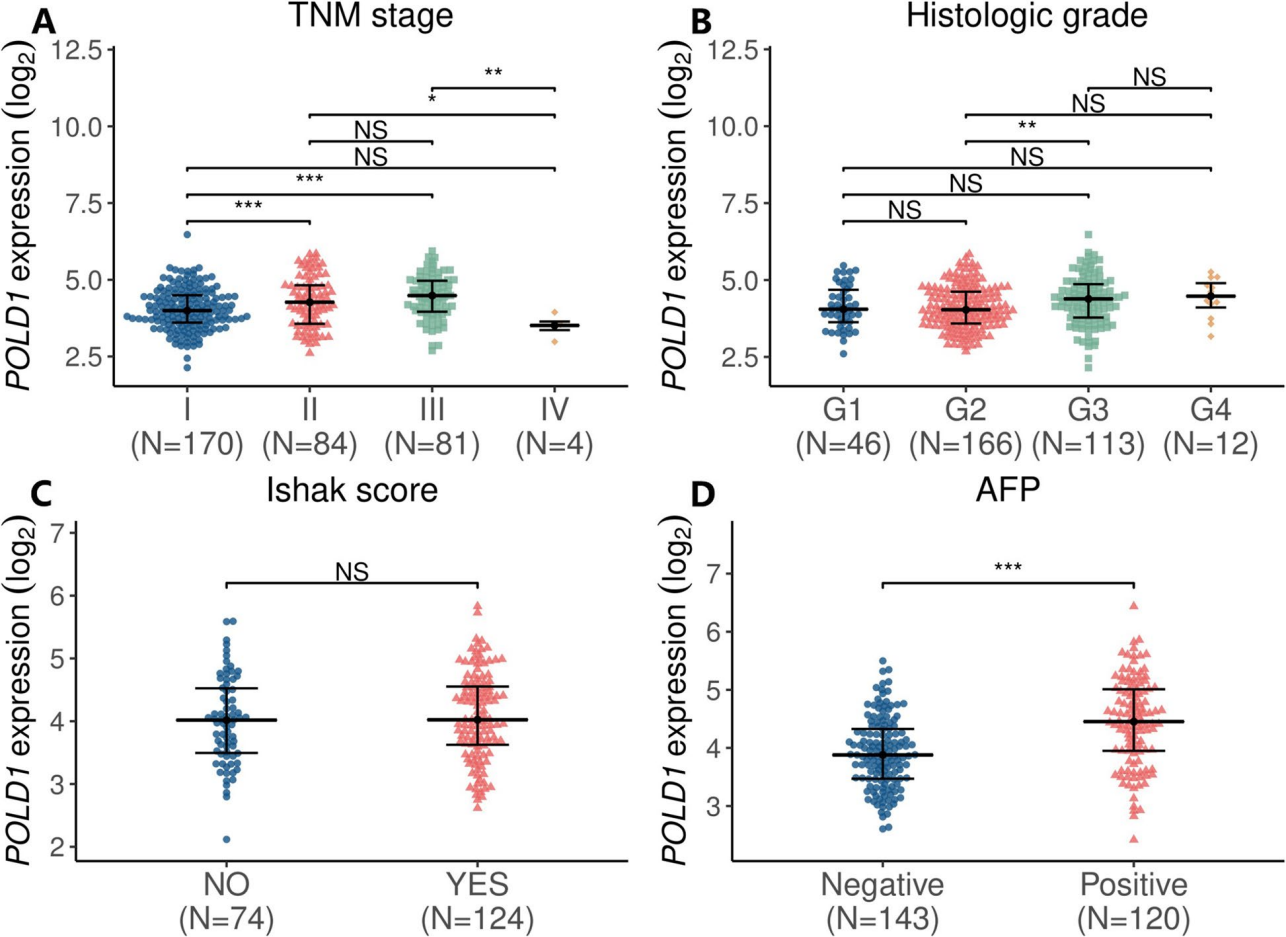
Comparison of POLD1 expression in different clinicopathological groups of HCC patients. Comparison of POLD1 expression in different groups of TNM stages **A**, histologic grades **B**, Ishak score **C**, and serum AFP **D**. (Quasi-likelihood F-test, *P *< 0.05 was considered significant, * P < 0.05, ** P < 0.01, *** *P* < 0.001)

The clinicopathological variables of 339 primary HCC patients retrieved from TCGA-LIHC were given in Table 1. Patients were classified into two groups (low/high) according to their POLD1 expression level and based on the optimal cut-off value of OS calculated by the “survminer” package of R software. As shown in Table 1; Fig. 2, POLD1 expression was not significantly associated with age, gender, Ishak score, Child − Pugh grade, family history of cancer, histologic grade, vascular invasion, and residual tumor (all P > 0.05). However, POLD1 upregulation was significantly correlated with positive alpha fetoprotein (AFP), and advanced TNM stage (all *P* < 0.05).

**Table 1:** Association between POLD1 expression and the clinicopathological variables in HCC patients (*n* = 339)

Variables	POLD1 Expression		P
	High (*n* = 172)	Low (*n* = 167)	
**Age (year)**			
< 65	112 (65.1%)	96 (57.5%)	0.183
≥ 65	60 (34.9%)	71 (42.5%)	
**Gender**			
Male	112 (65.1%)	119 (71.3%)	0.273
Female	60 (34.9%)	48 (28.7%)	
**Family history of cancer**			
No	104 (60.5%)	92 (55.1%)	0.153
Yes	42 (24.4%)	56 (33.5%)	
Unknown	26 (15.1%)	19 (11.4%)	
**TNM stage**			
I	71 (41.3%)	99 (59.3%)	**< 0.001**
II	47 (27.3%)	37 (22.2%)	
III	54 (31.4%)	27 (16.2%)	
IV	0 (0%)	4 (2.4%)	
**Histologic grade**			
G1–G2	88 (51.2%)	124 (74.3%)	**< 0.001**
G3–G4	83 (48.3%)	42 (25.1%)	
Unknown	1 (0.6%)	1 (0.6%)	
**Ishak score**			
0–4	61 (35.5%)	63 (37.7%)	**0.013**
5–6	28 (16.3%)	46 (27.5%)	
Unknown	83 (48.3%)	58 (34.7%)	
**Child–Pugh grade**			
A	103 (59.9%)	104 (62.3%)	0.331
B-C	8 (4.7%)	13 (7.8%)	
Unknown	61 (35.5%)	50 (29.9%)	
**Vascular invasion**			
None	93 (54.1%)	100 (59.9%)	0.230
Micro	41 (23.8%)	43 (25.7%)	
Macro	7 (4.1%)	7 (4.2%)	
Unknown	31 (18.0%)	17 (10.2%)	
**Alpha fetoprotein**			
Negative	49 (28.5%)	94 (56.3%)	**< 0.001**
Positive	82 (47.7%)	38 (22.8%)	
Unknown	41 (23.8%)	35 (21.0%)	
**Residual tumor**			
R0	151 (87.8%)	150 (89.8%)	0.761
R1-R2	6 (3.5%)	6 (3.6%)	
Unknown	15 (8.7%)	11 (6.6%)	

### POLD1 overexpression predicted poor OS in HCC

**Fig. 3:**
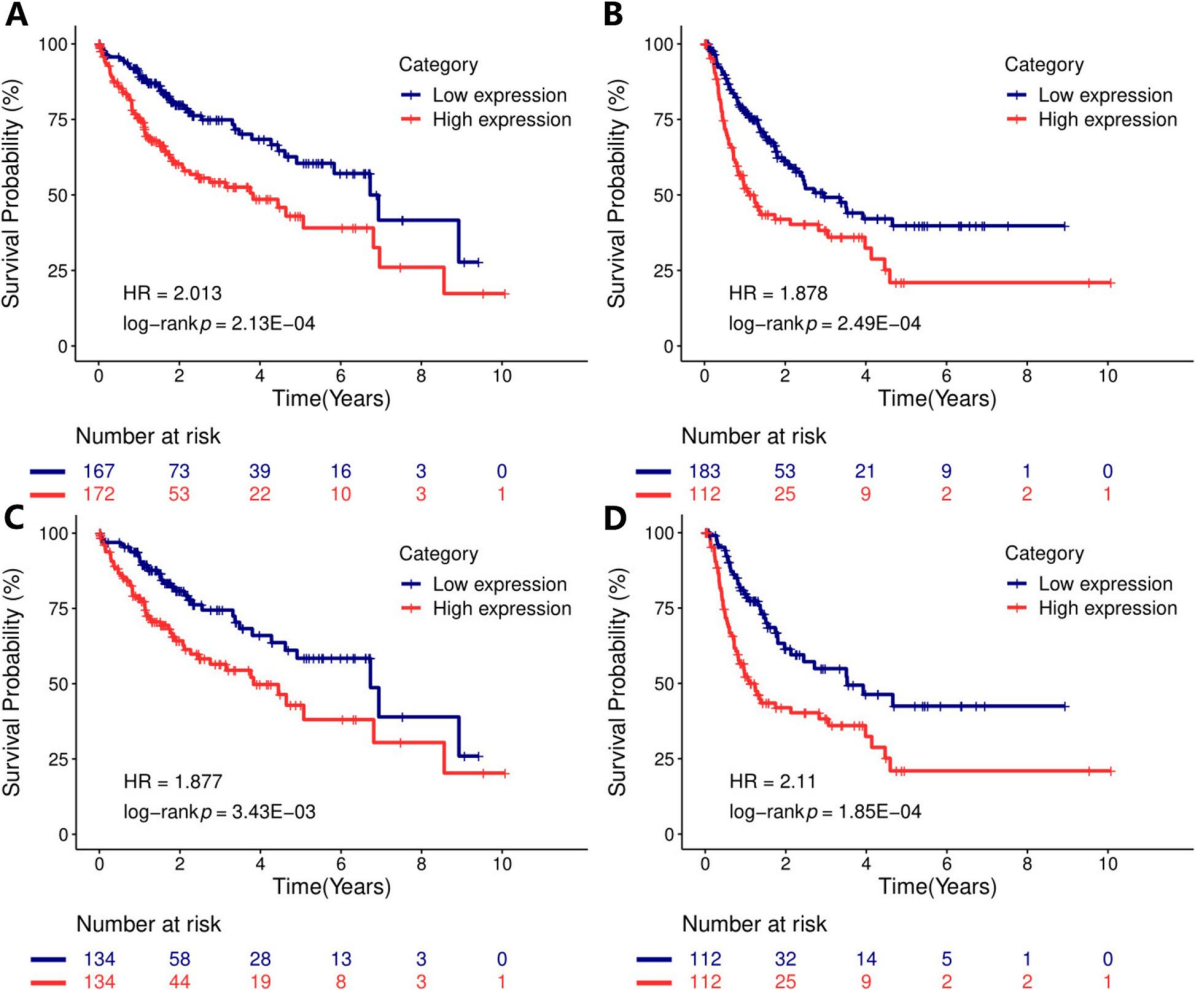
Kaplan-Meier curves of overall survival (**A**, **C**) and disease-free interval (**B**, **D**) for unpaired and matched HCC patients with high and low POLD1 mRNA levels using propensity-score matching analysis

The result of univariate analysis demonstrated that POLD1 mRNA level, TNM stage, and vascular invasion were significantly associated with the OS of HCC patients (Table 2). Further multivariate analysis confirmed that POLD1 overexpression (HR: 1.47, 95% CI: 1.1–1.97, *P* = 0.001) and advanced TNM stage were independent indicators of unfavorable OS (Table 2). As shown in Table 3, the univariate analysis demonstrated that POLD1 mRNA level was associated with the DFI of HCC patients. However, the multivariate analysis determined that POLD1 upregulation was not an independent indicator of poor DFI (HR: 1.29, 95% CI: 0.99–1.68, *P* = 0.057) (Table 3). Furthermore, Kaplan-Meier curves and log-rank tests were performed to show the effect of POLD1 expression on the OS and DFI of unpaired HCC patients (Fig. 3 A, B) and matched HCC patients after PSM analysis (Fig. 3 C, D).

**Table 2:** Univariate and multivariate analyses of overall survival

Variables	Univariate analysis		Multivariate analysis	
	HR (95% CI)	P	HR (95% CI)	P
Age (≥ 65 vs. < 65)	1.23(0.85,1.78)	0.273	-	-
Gender (Female vs. Male)	1.26(0.87,1.84)	0.228	-	-
Family history of cancer (Yes vs. No)	1.14(0.76,1.69)	0.53	-	-
TNM stage (II vs. I)	1.42(0.87,2.32)	0.16	1.21(0.64,2.28)	0.556
TNM stage (III vs. I)	2.72(1.78,4.15)	**< 0.001**	1.91(1.11,3.3)	**0.02**
TNM stage (IV vs. I)	5.44(1.68,17.63)	**0.005**	6.57(2,21.63)	**0.002**
Histologic grade (G3–G4 vs. G1–G2)	1.14(0.78,1.67)	0.489	-	-
Ishak score (5–6 vs. 0–4)	0.87(0.5,1.5)	0.612	-	-
Child–Pugh grade (B–C vs. A)	1.66(0.82,3.36)	0.159	-	-
Vascular invasion (Micro vs. None)	1.16(0.72,1.88)	0.539	1(0.57,1.75)	0.989
Vascular invasion (Macro vs. None)	2.52(1.14,5.58)	**0.023**	2.02(0.88,4.62)	0.097
Alpha fetoprotein (Positive vs. Negative)	1.45(0.92,2.28)	0.108	-	-
Residual tumor (R1–R2 vs. R0)	1.17(0.43,3.2)	0.754	-	-
POLD1 (High vs. Low)	1.58(1.23,2.02)	**< 0.001**	1.47(1.1,1.97)	**0.01**

**Table 3:** Univariate and multivariate analyses of disease-free interval

Variables	Univariate analysis		Multivariate analysis	
	HR (95% CI)	P	HR (95% CI)	P
Age (≥ 65 vs. < 65)	1.09(0.76,1.54)	0.65	-	-
Gender (Female vs. Male)	0.88(0.61,1.27)	0.487	-	-
Family history of cancer (Yes vs. No)	0.92(0.63,1.36)	0.683	-	-
TNM stage (II vs. I)	1.7(1.12,2.6)	**0.014**	1.78(1.05,3.02)	**0.032**
TNM stage (III vs. I)	2.84(1.89,4.26)	**< 0.001**	2.73(1.65,4.52)	**< 0.001**
TNM stage (IV vs. I)	-	-	-	-
Histologic grade (G3–G4 vs. G1–G2)	1.26(0.89,1.77)	0.197	-	-
Ishak score (5–6 vs. 0–4)	1.32(0.87,2.01)	0.196	-	-
Child–Pugh grade (B–C vs. A)	1.43(0.74,2.76)	0.287	-	-
Vascular invasion (Micro vs. None)	1.35(0.89,2.05)	0.161	0.92(0.56,1.5)	0.735
Vascular invasion (Macro vs. None)	2.82(1.34,5.92)	**0.006**	2.4(1.12,5.18)	**0.025**
Alpha fetoprotein (Positive vs. Negative)	1.08(0.74,1.59)	0.691	-	-
Residual tumor (R1–R2 vs. R0)	-	-	-	-
POLD1 (High vs. Low)	1.36(1.08,1.71)	**0.01**	1.29(0.99,1.68)	0.057

### Validation of the prognostic value of POLD1 based on nomogram

**Fig. 4:**
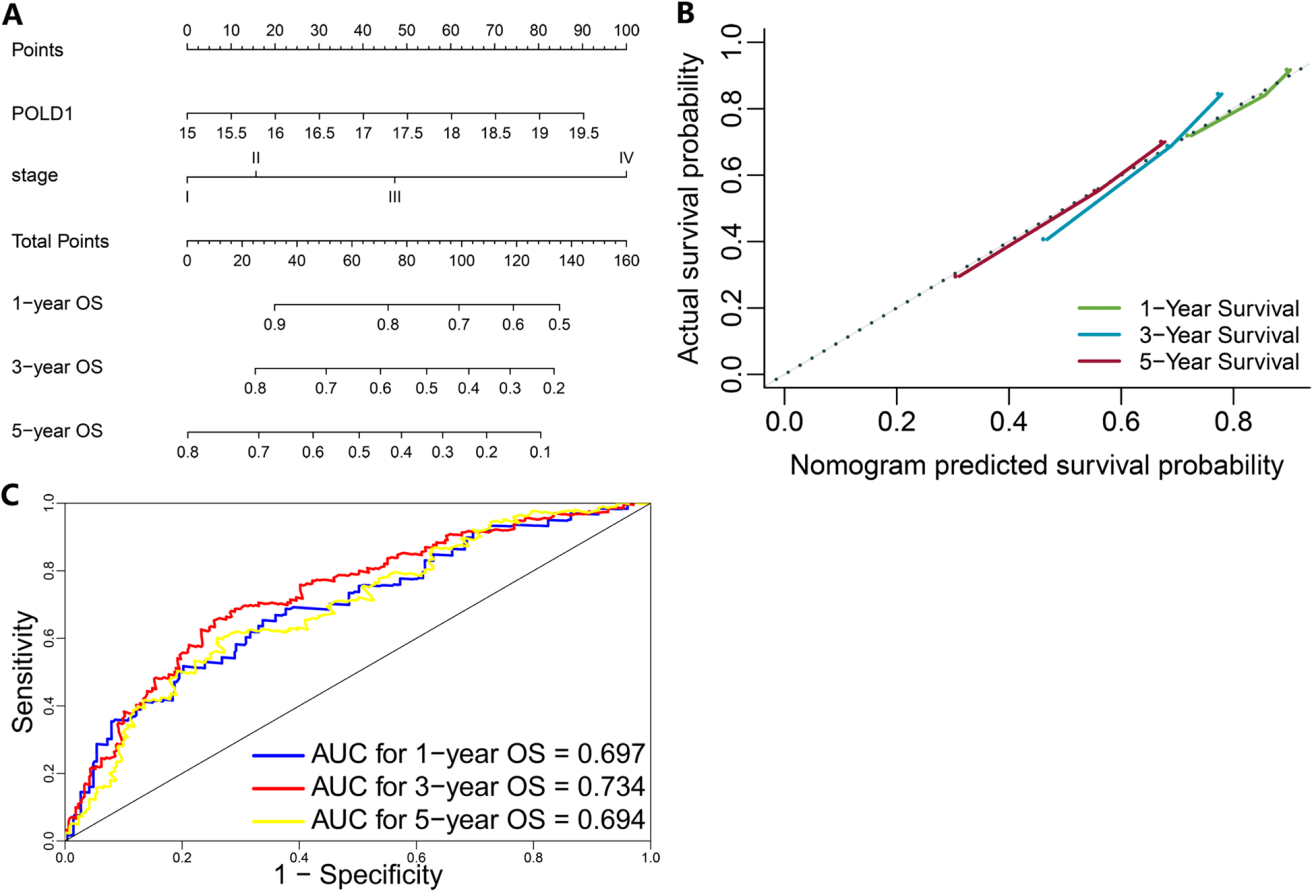
Validation of the prognostic value of POLD1 in postoperative HCC patients based on nomogram. The nomogram predicting overall survival (**A**). The calibration curve of the nomogram (**B**). The ROC curve of the nomogram (**C**)

Based on the result of multivariate Cox analyses (Table 2), the nomogram predicting the OS of HCC patients was constructed based on TNM stage and POLD1 mRNA expression level (Fig. 4 A). The C-index of the nomogram for OS prediction was 0.655 (95% CI: 0.627–0.684). As shown in the calibration plot (Fig. 4B), the nomogram demonstrated decent agreement between the predicted and actual survival outcome (1-, 3-, and 5-year OS). Besides, the AUC for 1-, 3-, and 5-year OS were 0.697, 0.734, and 0.694, respectively (Fig. 4 C).

### Validation of the prognostic value and clinical correlation of POLD1 using ICGC dataset

The predictive and prognostic value of POLD1 were examined in an independent external HCC dataset from the ICGC. Patients were classified into two groups (low/high) based on the median expression value of POLD1. As shown in Supplementary Figure S1A-D, the POLD1 mRNA was over-expressed in HCC samples (*n* = 207) compared with normal controls (*n* = 175) in ICGC-LIRI dataset. Moreover, POLD1 overexpression was correlated with advanced TNM stage and higher histologic grade. The Kaplan-Meier curve and log-rank test suggested POLD1 expression predicted poor OS in HCC patients from independent ICGC-LIRI dataset (*P* = 0.004) (Supplementary Figure S2).

### DNA copy gain, low POLD1 methylation and miR139-3p downregulation contributed to POLD1 overexpression in HCC

**Fig. 5:**
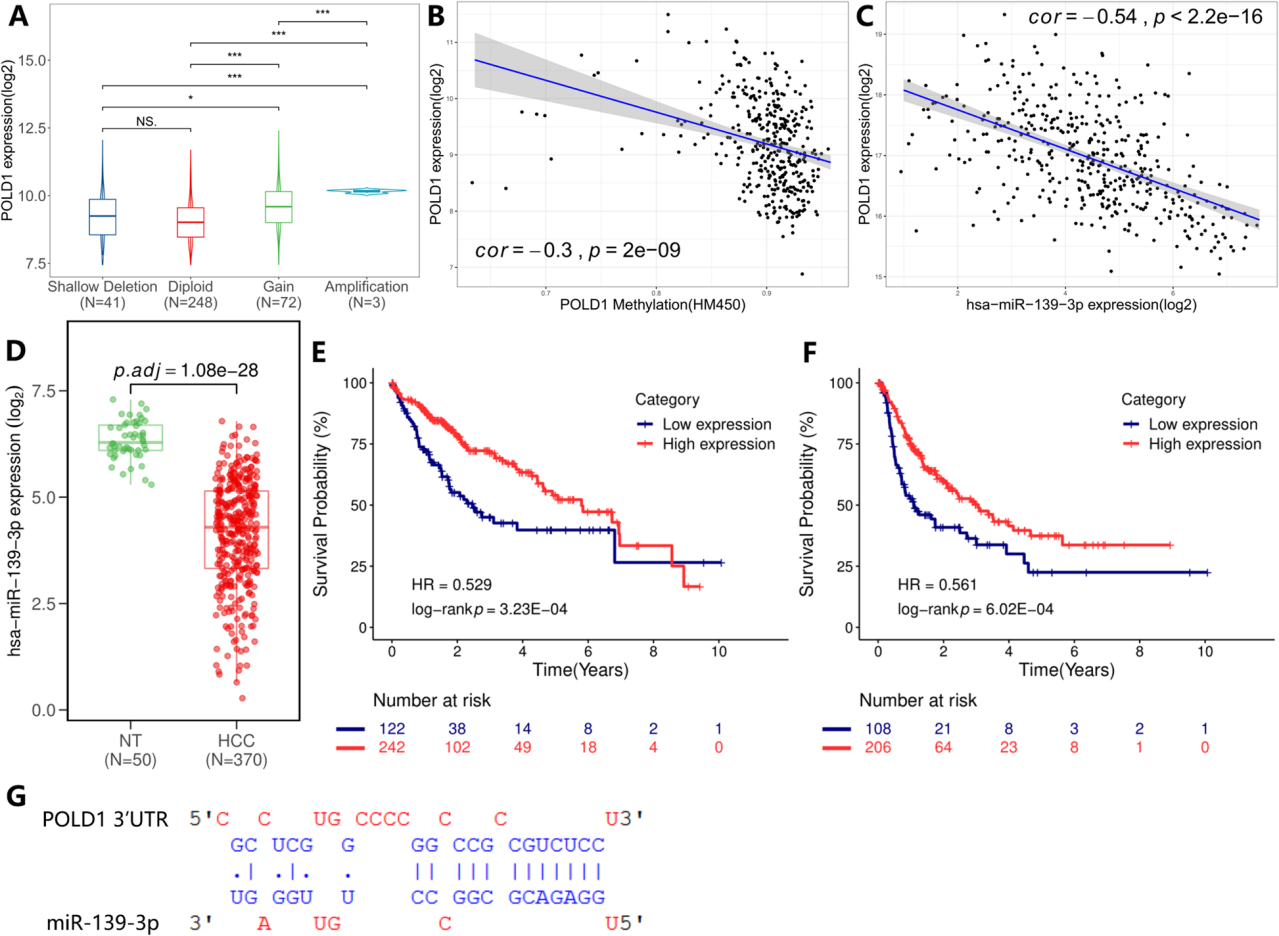
Exploration for underlying mechanisms of POLD1 overexpression in HCC. A Comparison of POLD1 mRNA expression in different copy number alteration (CNA) groups. **B** Correlation analysis between POLD1 DNA methylation and its mRNA expression. **C** Correlation analysis between miR-139-3p expression and POLD1 mRNA expression. **D** Comparison of miR-139-3p expression in HCC (*n* = 370) and in normal liver samples (*n* = 50). Kaplan-Meier curves of overall survival **E** and disease-free interval **F** for patients with high and low miR-139-3p expression levels. **G** The putative binding site of POLD1 3’ UTR by miR-139-3p. (Quasi-likelihood F-test, *P* < 0.05 was considered significant, * *P* < 0.05, ** *P* < 0.01, *** *P* < 0.001)

The underlying mechanisms of POLD1 overexpression in HCC carcinogenesis and development were further explored in terms of genetic and epigenetic alterations. There were 364 HCC patients with complete mRNA and CNA data in TCGA database, three patients had POLD1 amplification and 72 patients had POLD1 copy gain (low-level amplification). As shown in Fig. 5 A, POLD1 amplification and copy gain was significantly correlated with POLD1 mRNA upregulation (all *P* < 0.05). Besides, we examined the association between POLD1 DNA methylation and its mRNA expression. As shown in Fig. 5B, linear regression analysis demonstrated a significant negative correlation between POLD1 DNA methylation level and its mRNA expression (Pearson’s r = − 0. 3, *P* < 0.001).

Furthermore, we attempted to detect the regulatory miRNAs of POLD1. DIANA-microT database (http://diana.imis.athena-innovation.gr) was used to detect the regulatory miRNAs of POLD1 [21]. The common miRNAs detected by DIANA-microT and confirmed by TCGA-LIHC dataset were regarded as the potential regulatory miRNAs of POLD1 in HCC. As a result, miR-139-3p, miR-214-3p, miR-5589-3p, miR-195-5p, and miR-424-5p were considered as the potential regulatory miRNAs. MiR-214-3p, miR-5589-3p, miR-195-5p, and miR-424-5p expression were not associated with OS and/or DFI in HCC patients (data not shown). However, Kaplan-Meier curves suggested that low miR-139-3p expression was related to poor OS and DFI in patients from TCGA-LIHC dataset (all *P* < 0.001) (Fig. 5E, F). Therefore, miR-139-3p was selected for further exploration. As shown in Fig. 5 C, there was a significant negative correlation between the miR-139-3p and POLD1 mRNA expression (Pearson’s r = − 0.54, *P* < 0.001). Compared to adjacent normal liver samples, miR-139-3p was significantly downregulated in HCC tissues (*P* < 0.001) (Fig. 5D). The putative binding site of POLD1 3’ UTR by miR-139-3p retrieved from DIANA-microT was shown in Fig. 5G.

### POLD1 expression and immune cells infiltration analyses

**Fig. 6:**
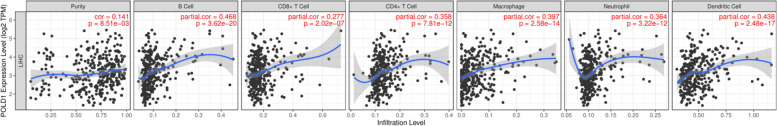
Correlations between POLD1 mRNA expression and six types of immune cell (B cell, CD4 + T cell, CD8 + T cell, macrophage, neutrophil, dendritic cell) infiltrated in HCC tissues (data were retrieved from TIMER2)

We also explored the association between POLD1 expression and immune cell infiltration in HCC tissues. As shown in Fig. 6, there were significant positive correlations between POLD1 mRNA expression and immune infiltration level of B cell (Pearson’s r = 0.468, *P* < 0.001), CD4 + T cell (Pearson’s r = 0.358, *P* < 0.001), macrophage (Pearson’s r = 0.397, *P* < 0.001), and dendritic cell (Pearson’s r = 0.438, *P* < 0.001). However, the correlation between POLD1 expression and CD8 + T Cell infiltration level (Pearson’s r = 0.277, *P* < 0.001) was weak.

### Co-expression genes and PPI network of POLD1

**Fig. 7:**
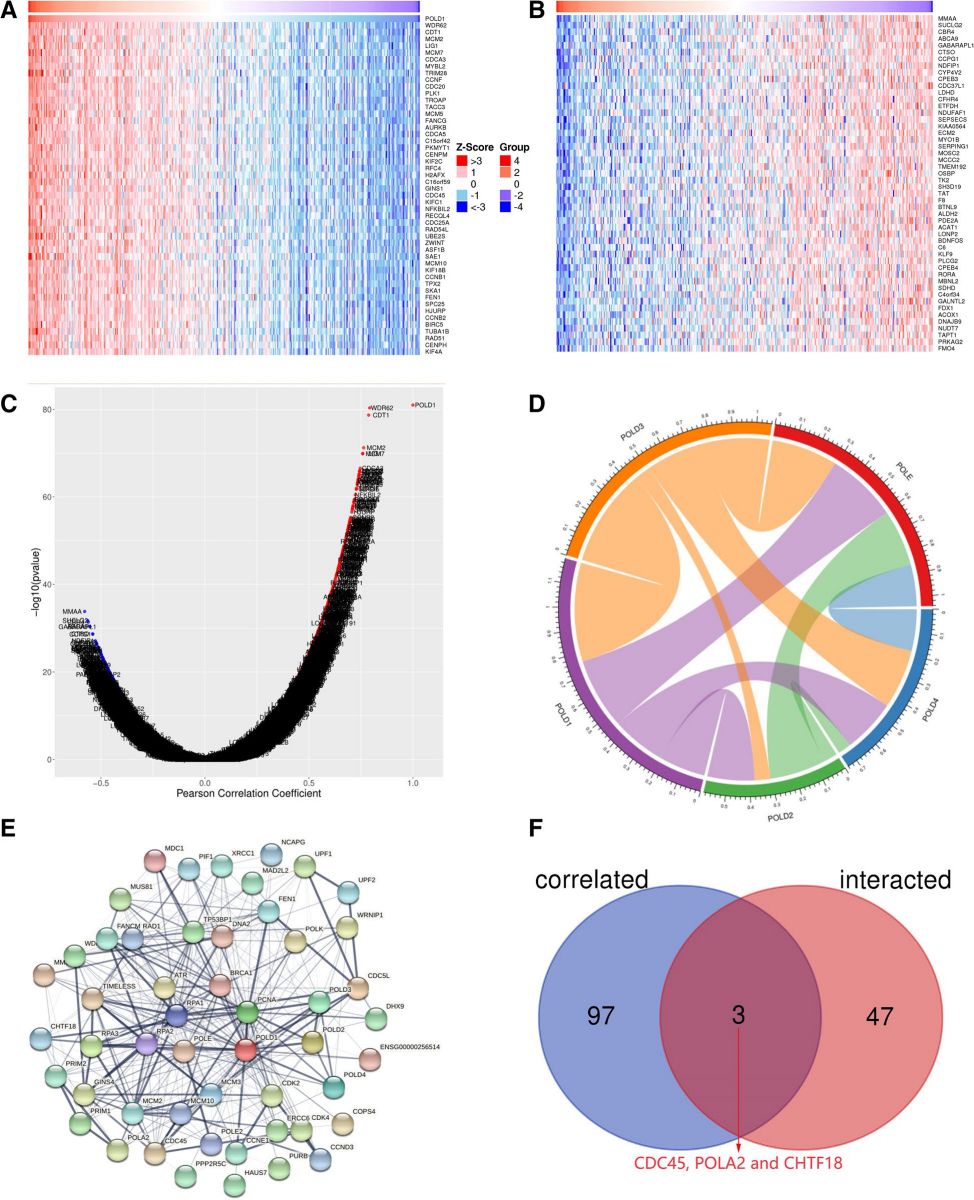
Genes co-expressed with POLD1 and interaction network of POLD1-binding proteins in HCC tissues. The top 50 significant genes positively (**A**) and negatively (**B**) correlated with POLD1. **C** The volcano plot showing the correlation between POLD1 and genes differentially expressed in HCC. **D** The chord diagram showing the correlation between POLD1 and POLD molecular family. **E** Interaction network of 50 POLD1-binding proteins. **F** An intersection analysis of the POLD1-binding and correlated genes

**Fig. 8:**
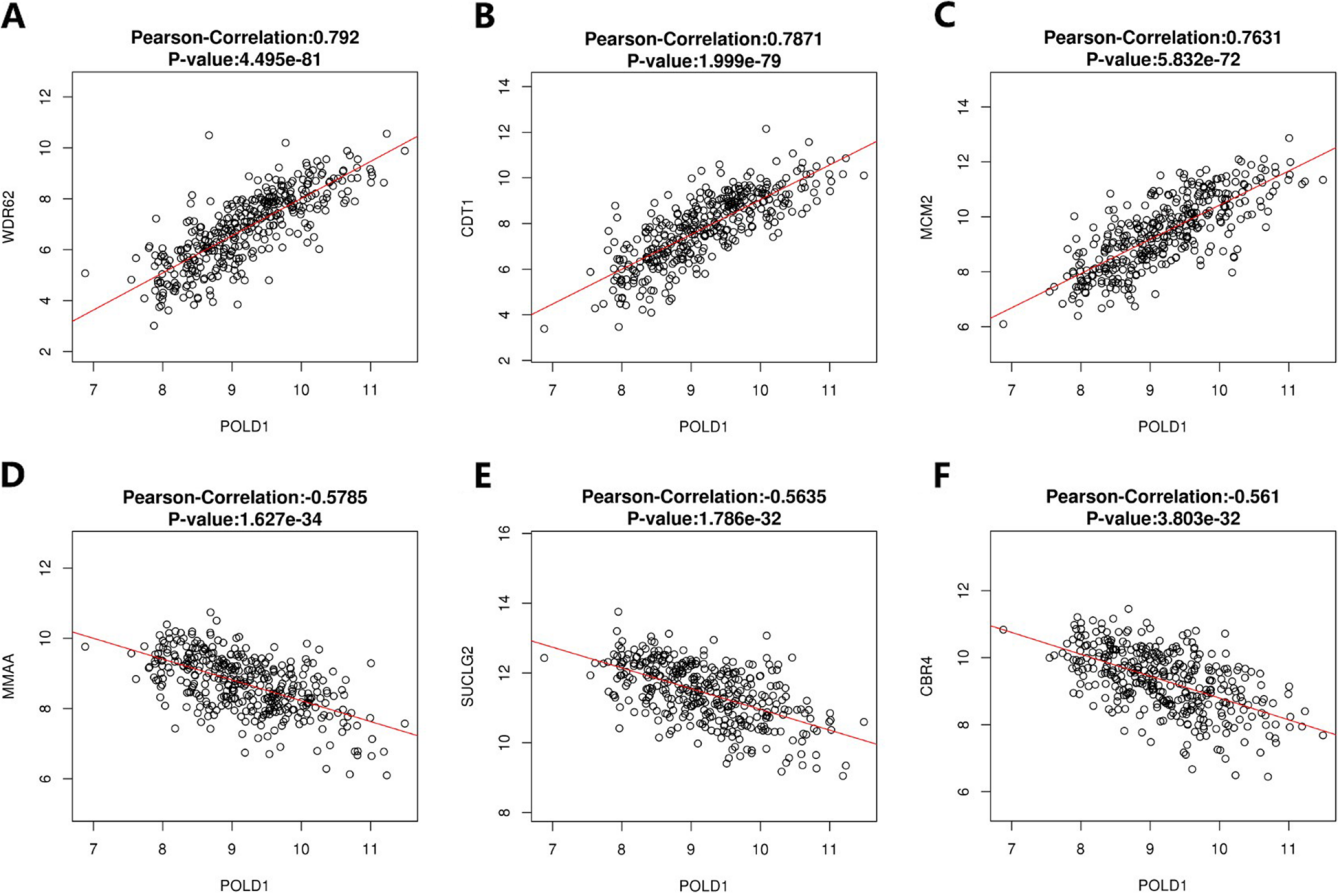
Correlation analysis between POLD1 mRNA expression and the top three differentially expressed genes in HCC (*n* = 371). **A**–**C** The positive correlation between POLD1 and the top three genes (WDR62, CDT1 and MCM2). **D**–**F** The negative correlation between POLD1 and the top three genes (MMAA, SUCLG2 and CBR4)

Genes co-expressed with POLD1 were retrieved from LinkedOmics, which analyzed mRNA sequencing data of 371 patients from TCGA-LIHC dataset. The heatmaps (Fig. 7 A, B) showed the top 50 significant genes positively and negatively correlated with POLD1. The volcano plot (Fig. 7 C) showed all the genes associated with POLD1. While WDR62, CDT1 and MCM2 were the top three genes positively correlated with POLD1 mRNA expression (Fig. 8 A-C), MMAA, SUCLG2 and CBR4 were the top three genes negatively correlated with POLD1 mRNA expression (Fig. 8D-F). Moreover, we performed Pearson correlation analysis to examine the relationship between POLD1 and POLD molecular family, as well as DNA polymerase epsilon (POLE) (Fig. 7D). Furthermore, we obtained 50 POLD1-binding proteins from STRING, which were supported by experimental evidence. Figure 7E showed the interaction network of these proteins. Figure 7 F showed the intersection analysis of the top 100 POLD1-correlated genes retrieved from GEPIA2 and 50 POLD1-binding proteins obtained from STRING, demonstrating the three common members (CDC45, POLA2 and CHTF18) among the above two datasets.

### Functional enrichment analysis of POLD1 in HCC

**Fig. 9:**
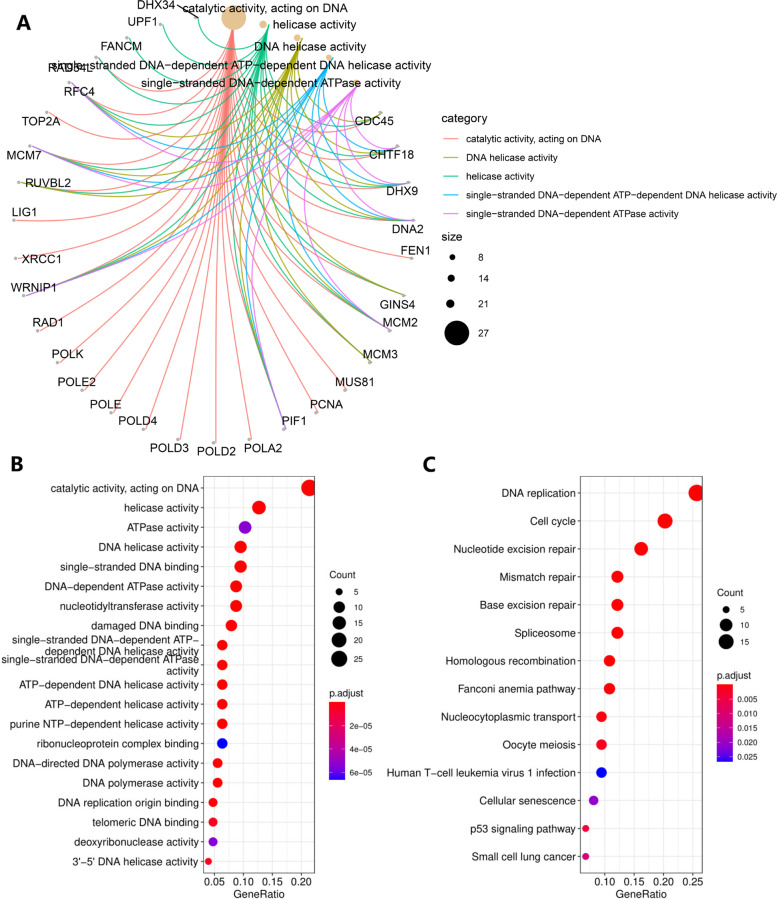
Functional enrichment analysis of POLD1 in HCC. **A** The cnetplot for the molecular function data in GO analysis. **B** The significantly enriched GO annotations. **C** The KEGG pathway enrichment analysis

GO and KEGG pathway enrichment analyses were performed with the combination of 100 POLD1-correlated genes and 50 POLD1-binding proteins. The GO analysis demonstrated that most of these genes were linked to the pathways or cellular biology of DNA replication (such as helicase activity, DNA polymerase activity), DNA damage repair (such as damaged DNA binding) and DNA metabolism (such as catalytic activity acting on) (Fig. 9 A, B). The KEGG analysis demonstrated “DNA replication”, “nucleotide excision repair” and “cell cycle” pathways might be involved in the effect of POLD1 on HCC pathogenesis (Fig. 9 C).

### Effect of POLD1 mRNA expression on prognosis and genomic stability in pan-cancer

We further evaluated the effect of POLD1 mRNA expression on the prognosis of patients with various cancer types. To simplify the research work, patients were classified into two groups (low/high) based on the median expression value of POLD1. As shown in Supplementary Figure S3A, univariate Cox analysis suggested that POLD1 mRNA level was associated with the OS of patients with adrenocortical carcinoma (ACC, HR: 5.31, 95% CI: 2.84–9.93, *P* < 0.001), diffuse large B-cell lymphoma (DLBC, HR: 0.18, 95% CI: 0.03–0.99, *P* = 0.049), kidney chromophobe (KICH, HR: 5.79, 95% CI: 1.45–23.13, *P* = 0.013), clear cell renal cell carcinoma (KIRC, HR: 2.46, 95% CI: 1.73–3.51, *P* < 0.001), brain lower grade glioma (LGG, HR: 1.93, 95% CI: 1.53–2.44, *P *< 0.001), mesothelioma (MESO, HR: 2.97, 95% CI: 1.87–4.72, *P* < 0.001), pheochromocytoma and paraganglioma (PCPG, HR: 9.25, 95% CI: 1.47–58.34, *P* = 0.018), prostate adenocarcinoma (PRAD, HR: 3.65, 95% CI: 1.33-10, *P* = 0.012), sarcoma (SARC, HR: 1.68, 95% CI: 1.22–2.31, *P* = 0.001), uveal melanoma (UVM, HR: 0.11, 95% CI: 0.02–0.46, *P* = 0.003), and HCC (HR: 1.65, 95% CI: 1.29–2.11, *P* < 0.001). Moreover, POLD1 mRNA level was significantly associated with the DFI of patients with PRAD (HR: 4.73, 95% CI: 2.14–10.41, *P* < 0.001), SARC (HR: 1.61, 95% CI: 1.12–2.32, *P* = 0.011), and HCC (HR: 1.29, 95% CI: 1.06–1.58, *P* = 0.011) (Supplementary Figure S3B). However, Kaplan-Meier curve and log-rank test suggested there was no significant association between POLD1 expression and the DFI of HCC (*P* = 0.078). Kaplan-Meier curves and log-rank tests were performed to show the effect of POLD1 expression on the OS and DFI of patients with various cancer types (Supplementary Figure S4 and S5).

TMB and MSI are considered to be important factors impacting carcinogenesis and survival. We determined the relationship between TMB or MSI and POLD1 mRNA expression in pan-cancer. The results suggested that POLD1 expression was significantly positively correlated with TMB in ACC, bladder urothelial carcinoma (BLAC), breast invasive carcinoma (BRCA), glioblastoma multiforme (GBM), KICH, LGG, lung adenocarcinoma (LUAD), lung squamous cell carcinoma (LUSC), MESO, ovarian serous cystadenocarcinoma (OV), pancreatic adenocarcinoma (PAAD), SARC, skin cutaneous melanoma (SKCM), stomach adenocarcinoma (STAD), testicular germ cell tumor (TGCT), thyroid carcinoma (THCA), uterine corpus endometrial carcinoma (UCEC), and uterine carcinosarcoma (UCS) (Supplementary Figure S3C). While increased POLD1 expression was significantly associated with decreased TMB in thymoma (THYM) (Supplementary Figure S3C). Likewise, POLD1 expression was significantly positively correlated with MSI in ACC, BLCA, BRCA, CESC, head and neck squamous cell carcinoma (HNSC), KICH, KIRC, HCC, LUAD, LUSC, PRAD, SARC, STAD, THCA, and UCEC (Supplementary Figure S3D). While increased POLD1 expression was significantly associated with decreased TMB in rectum adenocarcinoma (READ) (Supplementary Figure S3D).

## Discussion

HCC accounts for approximately 80% of liver cancers, which is the sixth most common cancer and the fourth leading cause of cancer-related death worldwide [22]. With a 5-year survival of 18% [1], it is needed to detect more novel diagnostic and therapeutic targets of HCC, which will promote early diagnosis and personalized tumor management.

POLD1 plays a key role in cell cycle and DNA repair [23]. Previous studies suggested that POLD1 proofreading domain mutations were identified as predisposing to a range of cancers, including colorectal cancer, endometrial cancer, skin squamous cell carcinoma, breast cancer, and brain tumor [6–8]. Moreover, POLD1 upregulation contributes to cancer cell proliferation, migration, and invasion, as well as survival under replication stress via improving their tolerance to DNA damage [23, 24]. It has been proposed that an elevated level of DNA polymerase delta may favor late-stage oncogenesis [23].

Although studies have confirmed that ICIs-containing treatment regimens can improve the survival of patients with HCC, not all patients can benefit from ICIs [25]. Therefore, patient selection using predictive biomarkers would be desirable, and it has been proved that TMB and MSI status may be promising biomarkers [25]. Considering the role of POLD1 in DNA repair, it is understandable that POLD1 proofreading domain mutations, even non-proofreading mutations, are significantly correlated with higher TMB and high MSI (MSI-H) status [9, 10]. Higher TMB and MSI-H status were believed to be associated with improved survival in patients treated with ICIs across multiple cancer types [26, 27]. Further, Wang et al. [10] suggested that POLE/POLD1 mutations could potentially predict desirable outcomes in patients treated with ICIs across multiple cancer types, even if cancer type and MSI status were adjusted. Moreover, the defective POLD1 function caused by its mutation has been regarded as a cause of T cell immunodeficiency [28]. Intriguingly, siRNA-mediated POLD1 depletion impaired proliferation and/or invasive potential, and increased genome instability of (liver, breast, cervical and osteosarcoma) cancer and normal cells [5, 29, 30], which confirmed the key role of POLD1 in cell cycle progression and DNA damage repair. The relationship between two genes, in which single mutations alone are compatible with cell survival, but mutation of both leads to death, is defined as synthetic lethality [31]. Based on this concept, multiple studies demonstrated that colorectal cancer with deficient POLD1 activity possessed the increased sensitivity to ATR and CHK1 inhibitors in preclinical models [32, 33]. However, the effect and underlying mechanisms of POLD1 in HCC are not well-understood.

In the current study, using data from TCGA-LIHC and the HPA, we observed that POLD1 was over-expressed in HCC than in normal liver samples at mRNA and protein levels, which was verified by another independent research [5]. Moreover, POLD1 upregulation was an independent indicator of poor OS, but not DFI, and was correlated with positive AFP and advanced TNM stage. These findings, including the association between POLD1 mRNA expression and TNM stage, as well as the prognostic value of POLD1, were verified using an independent validation cohort from ICGC database.

DNA methylation is the most intensively studied epigenetic mechanism, dysregulated DNA methylation is associated with HCC pathogenesis and progression, and plays role in increased chromosomal instability [34]. While miRNAs are the most well-studied epigenetic regulators in liver cancer, and were reported to be associated with HCC progression and prognosis [34]. Here, we attempted to analyze the mechanisms of the aberrantly expressed POLD1 in HCC and observed that DNA copy gain, low POLD1 methylation, and miR-139-3p downregulation may contribute to POLD1 overexpression. The putative binding site of POLD1 3’ UTR by miR-139-3p supported that miR-139-3p might be one of the regulators of POLD1 in HCC. A previous study reported that miR-155 is one of the regulatory miRNAs of POLD1 in a mouse model [35], whereas this finding is not confirmed in HCC by our study (data not shown).

To further identify the underlying mechanisms of POLD1 in HCC carcinogenesis and development, we assessed the association between POLD1 expression and immune cells infiltration. Co-expression genes, PPI network, and functional enrichment analyses of POLD1 in HCC were also examined. We confirmed that there were significant positive correlations between POLD1 mRNA expression and immune infiltration level of B cell, CD4 + T cell, macrophage, neutrophil and dendritic cell. Moreover, several studies reported that the higher immune infiltration level of dendritic cell, macrophage, and tumor-infiltrating lymphocyte may be predictive biomarkers for ICIs therapy [36, 37]. Furthermore, we identified the top 100 POLD1-correlated genes and 50 experimentally determined POLD1-binding proteins, functional enrichment analysis demonstrated “DNA replication”, “mismatch repair” and “cell cycle” pathways might be involved in the effect of POLD1 on HCC pathogenesis. The top three genes (WDR62, CDT1 and MCM2) positively correlated with POLD1 expression play important roles in cell proliferation and apoptosis, and their upregulation has been proved to be associated with tumors development and worse prognosis, such as breast cancer and lung adenocarcinoma [38–40]. This is in accordance with the result of the functional enrichment analysis of POLD1 in HCC. However, the role of the top three genes (MMAA, SUCLG2 and CBR4) negatively correlated with POLD1 expression in cancers is not elucidated.

Unfortunately, we were unable to analyze the effect of POLD1 expression on HCC patients treated with ICIs, because there was no data on HCC patients treated with ICIs in TCGA-LIHC dataset. Besides, we failed to analyze the effect of POLD1 mutation on HCC pathogenesis and patients’ survival, because there were only 4 patients with POLD1 mutation among 373 HCC patients in TCGA-LIHC dataset, and no patient carried POLD1 proofreading domain mutation (data not shown).

To verify the prognostic value of POLD1 in patients with various cancer types, Cox analysis and Kaplan-Meier curves were performed in pan-cancer. The results suggested that POLD1 overexpression was correlated with the poor OS of patients with ACC, KICH, KIRC, LGG, MESO, PCPG, PRAD, SARC, and HCC. Moreover, POLD1 overexpression was correlated with the poor DFI of patients with PRAD and SARC. As a supplement to previous studies [9, 10], our results suggested that POLD1 overexpression was significantly correlated with higher TMB in ACC, BLAC, BRCA, GBM, KICH, LGG, LUAD, LUSC, MESO, OV, PAAD, SARC, SKCM, STAD, TGCT, THCA, UCEC, and UCS. Moreover, POLD1 expression was significantly positively correlated with MSI in ACC, BLCA, BRCA, CESC, HNSC, KICH, KIRC, HCC, LUAD, LUSC, PRAD, SARC, STAD, THCA, and UCEC. These results further confirmed the role of POLD1 in cancer prognosis and genomic instability, and POLD1 could be a promising prognostic marker and potential therapeutic target in various cancers, or biomarker for response to ICIs therapy.

There were still some limitations in our study though we explored the effect and underlying mechanisms of POLD1 in HCC pathogenesis from several different perspectives. First, as mentioned above, we failed to analyze the effect of POLD1 expression on HCC patients treated with ICIs, and the effect of POLD1 mutation on HCC due to its rarity. Second, although the sample size of TCGA-LIHC was relatively large, and the prognostic value of POLD1 was verified using an external validation cohort, further preclinical mechanistic studies and prospective clinical trials are still needed to explore and validate the role of POLD1 in HCC. Several phase 2 clinical trials (such as NCT03461952, NCT02693535, NCT03428802, and NCT03207347) are ongoing to evaluate the efficacy of ICIs or targeted therapies in patients with cancer and POLE/POLD1 mutations. The results of the above clinical trials and more research on the role of POLD1 in cancer carcinogenesis and development would reach more instructive conclusions and contribute to personalized tumor management.

## Conclusions

In conclusion, we found that POLD1 is significantly upregulated in HCC tissue. Overexpression of POLD1 promoted HCC progression potentially through accelerating cell-cycle and improving tolerance of tumor cells to DNA damage. These results help elucidate molecular pathways of HCC carcinogenesis and development. Meanwhile, POLD1 may be a potential prognostic marker and promising therapeutic target in HCC and various cancers.

## Supplementary Information

**Additional file 1: Figure S1.** (A) Comparison of POLD1 mRNA expression in HCC (*n* = 207) and in normal liver tissues (*n* = 175) in ICGC-LIRI dataset. Comparison of POLD1 expression in different groups of living status (B), TNM stages (C), and histologic grades (D). (Quasi-likelihood F-test, P< 0.05 was considered significant, * *P *< 0.05, ** *P *< 0.01, *** *P *< 0.001).** Figure S2**. Kaplan-Meier curves of overall survival for HCC patients with high and low POLD1 mRNA levels from ICGC-LIRI dataset.** Figure S3**. Effect of POLD1 expression on prognosis and genomic stability in TCGA pan-cancer cohort. Univariate Cox analysis showed the association between POLD1 expression and overall survival (A) or disease-free interval (B). Correlation between POLD1 expression and TMB (C) or MSI (D). Spearman’s correlation coefficients are shown above the bar graphs. (Spearman correlation test, P< 0.05 was considered significant, * *P *< 0.05, ** *P *< 0.01, *** *P *< 0.001).** Figure S4**. Kaplan-Meier curves and log-rank tests for overall survival of patients with high and low POLD1 mRNA levels in adrenocortical carcinoma (A), diffuse large B-cell lymphoma (B), clear cell renal cell carcinoma (C), kidney renal papillary cell carcinoma (D), brain lower grade glioma (E), HCC (F), lung adenocarcinoma (G), mesothelioma (H), sarcoma (I), and uveal melanoma (J).** Figure S5**. Kaplan-Meier curves and log-rank tests for disease-free interval of patients with high and low POLD1 mRNA levels in prostate adenocarcinoma (A), and sarcoma (B).

## Data Availability

The datasets generated and/or analyzed in the current study are available in the TCGA database (http://cancergenome.nih.gov) and the ICGC database (http://icgc.org).

## Abbreviations

ACC: Adrenocortical carcinoma
AFP: Alpha fetoprotein
AUC: Area under the curve
BRCA: Breast invasive carcinoma
C-index: Concordance index
CI: Confidence interval
CNA: Copy number alterations
DFI: Disease-free interval
DLBC: Diffuse large B-cell lymphoma
FC: Fold change
GBM: Glioblastoma multiforme
GO: Gene ontology
HCC: Hepatocellular carcinoma
HNSC: Head and neck squamous cell carcinoma
HPA: Human Protein Atlas
HR: Hazard ratio
ICGC: International Cancer Genome Consortium
ICIs: Immune-checkpoint inhibitors
IHC: Immunohistochemistry
KEGG: Kyoto encyclopedia of genes and genomes
KICH: Kidney chromophobe
KIRC: Kidney clear cell renal cell carcinoma
LIHC: Liver hepatocellular carcinoma
LIRI: Liver cancer - RIKEN
LGG: Lower grade glioma
LUAD: Lung adenocarcinoma
LUSC: Lung squamous cell carcinoma
MESO: Mesothelioma
MSI: Microsatellite instability
MSI-H: High MSI
