# Supplementary figures and images for: A Comprehensive Prognostic Analysis of POLD1 in Hepatocellular Carcinoma
Source: BMC Cancer. 2022 Feb 21;22:197. doi: 10.1186/s12885-022-09284-y (PMC8862270; doi:10.1186/s12885-022-09284-y)

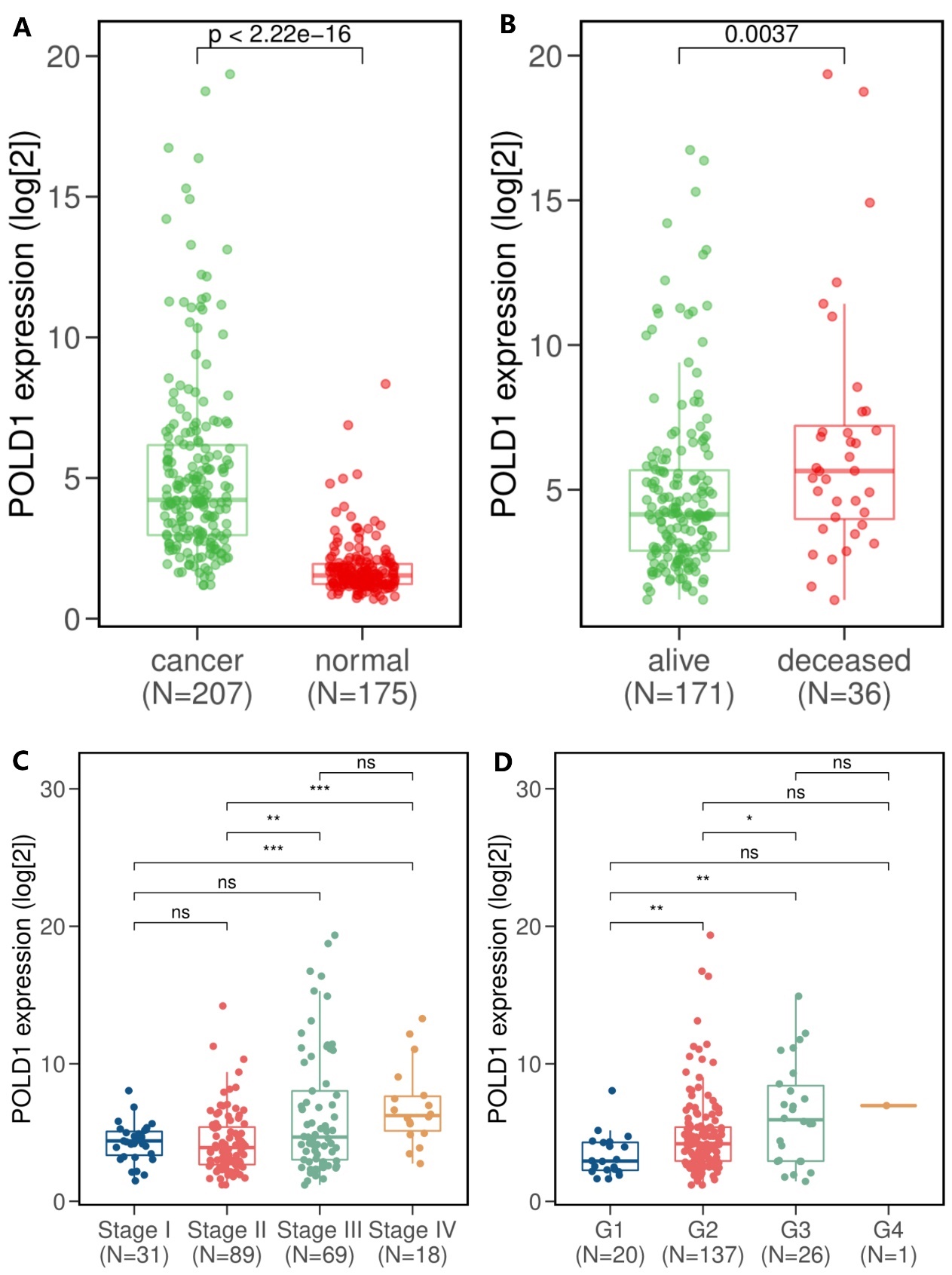


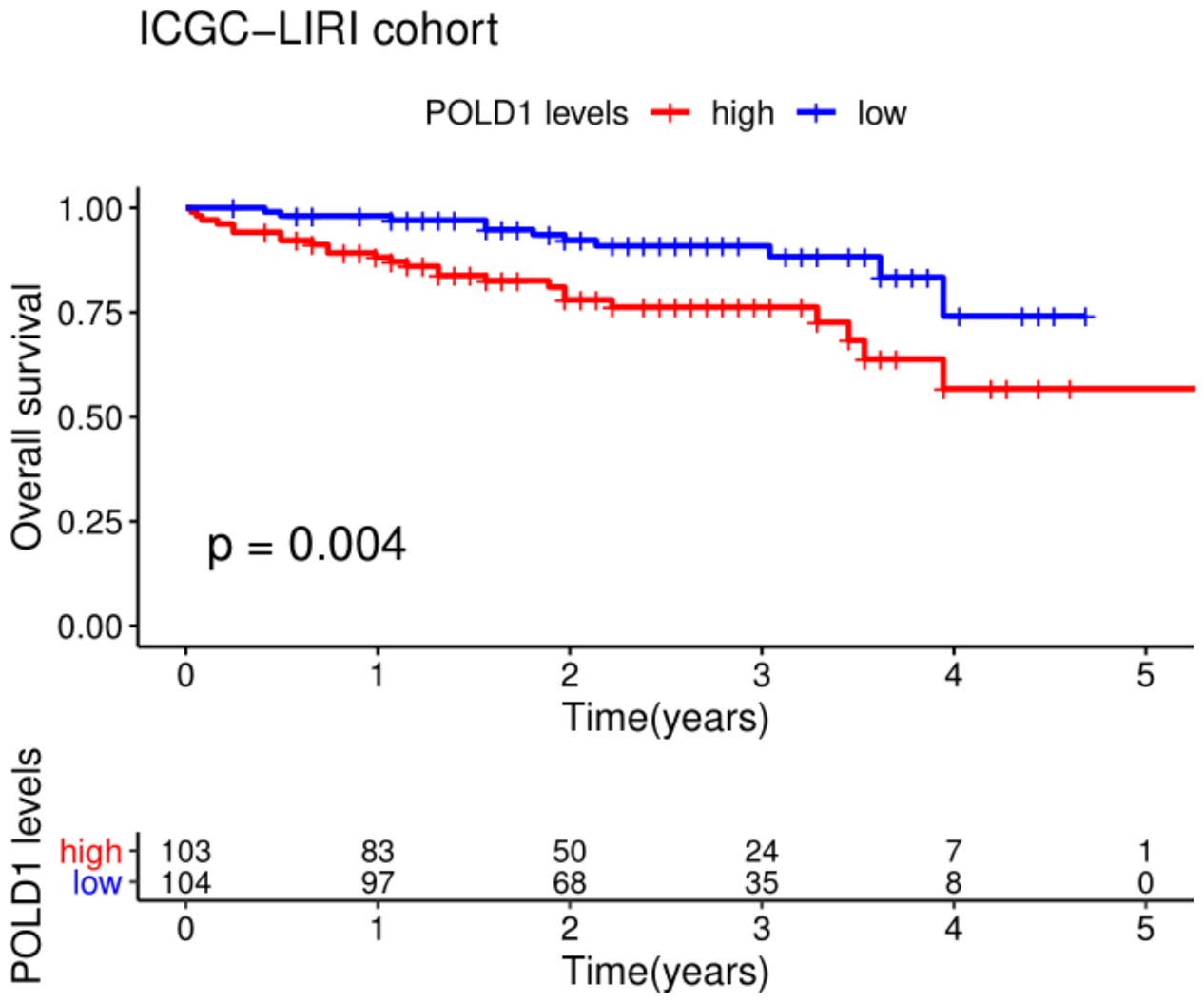


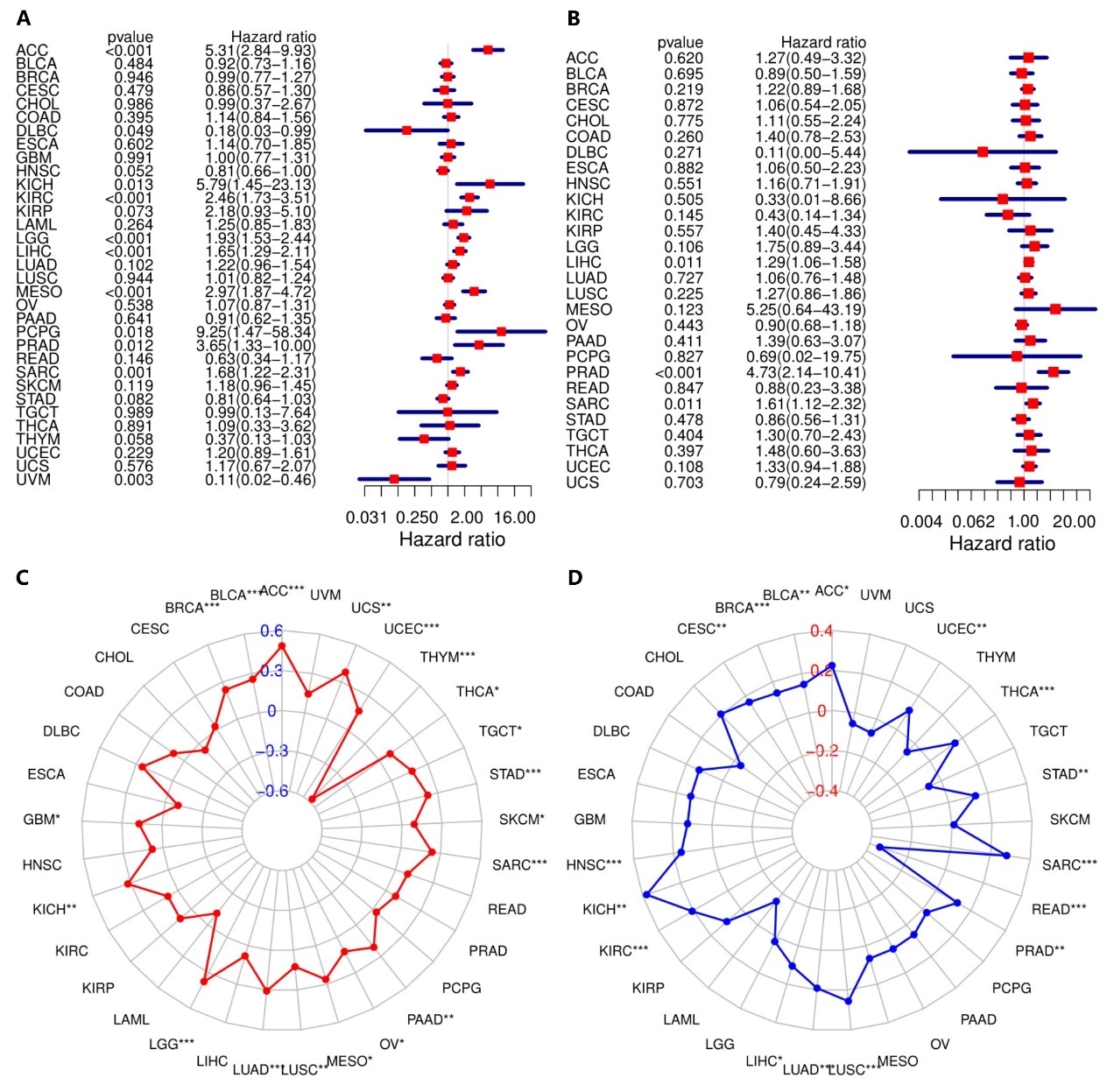


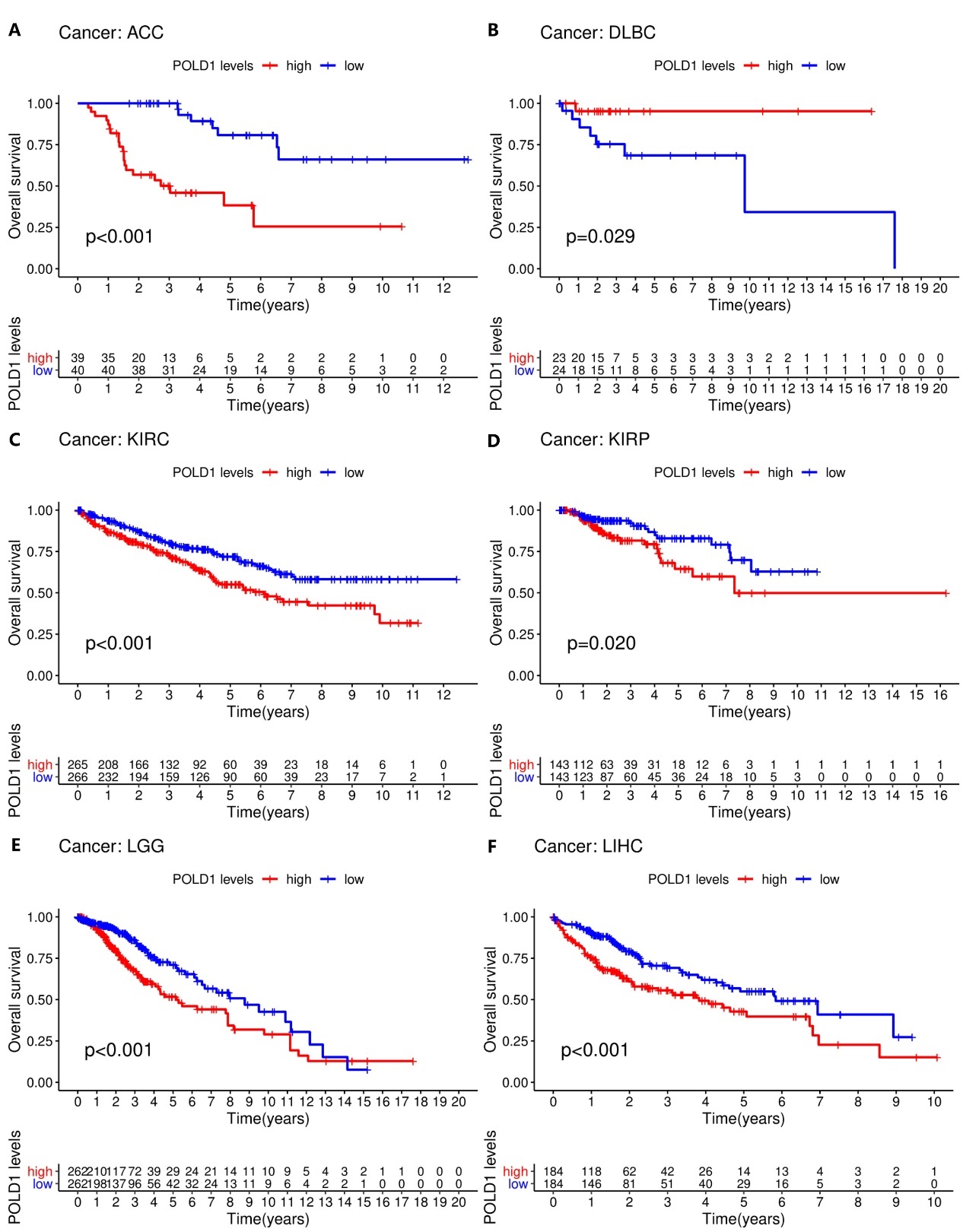


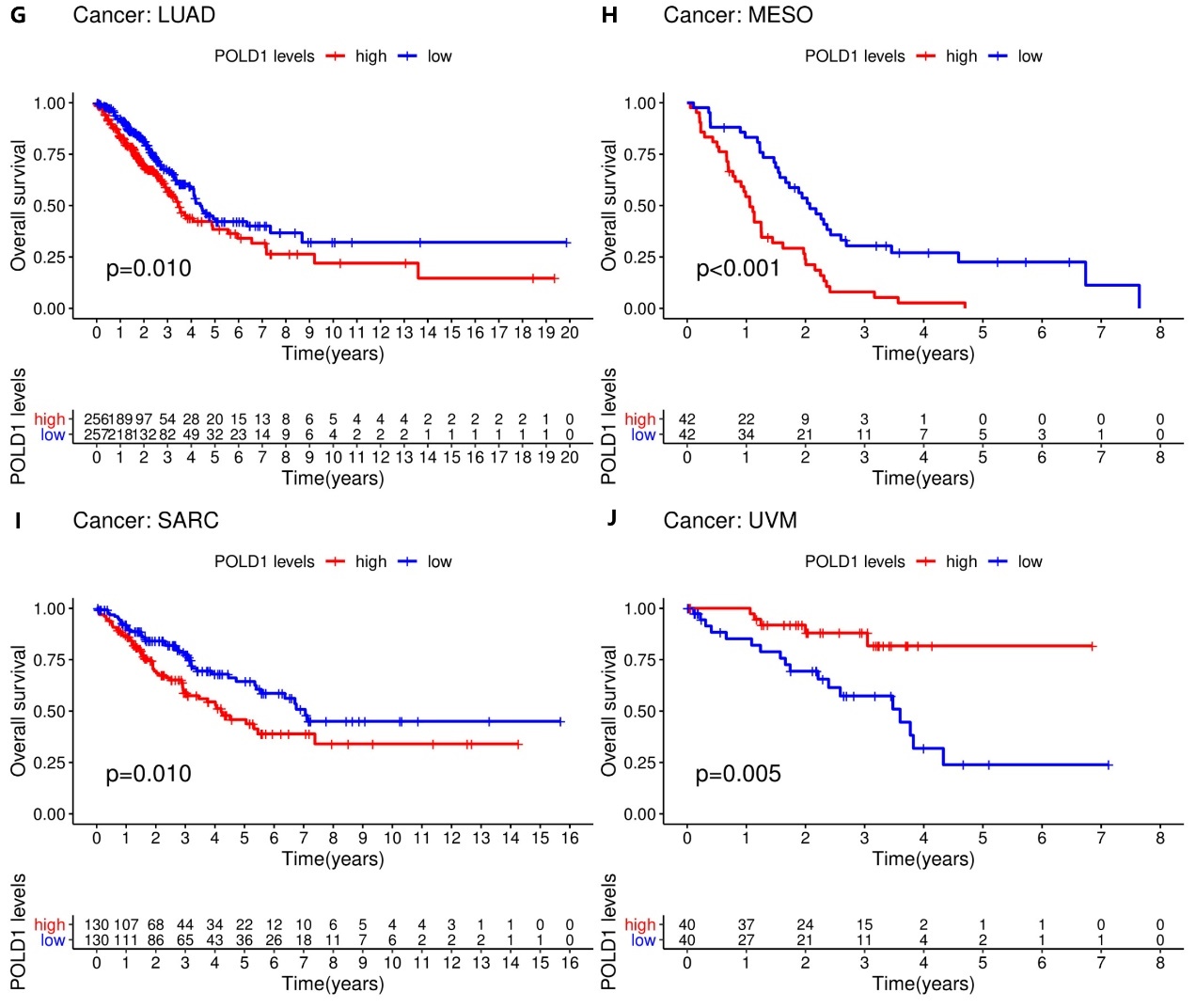


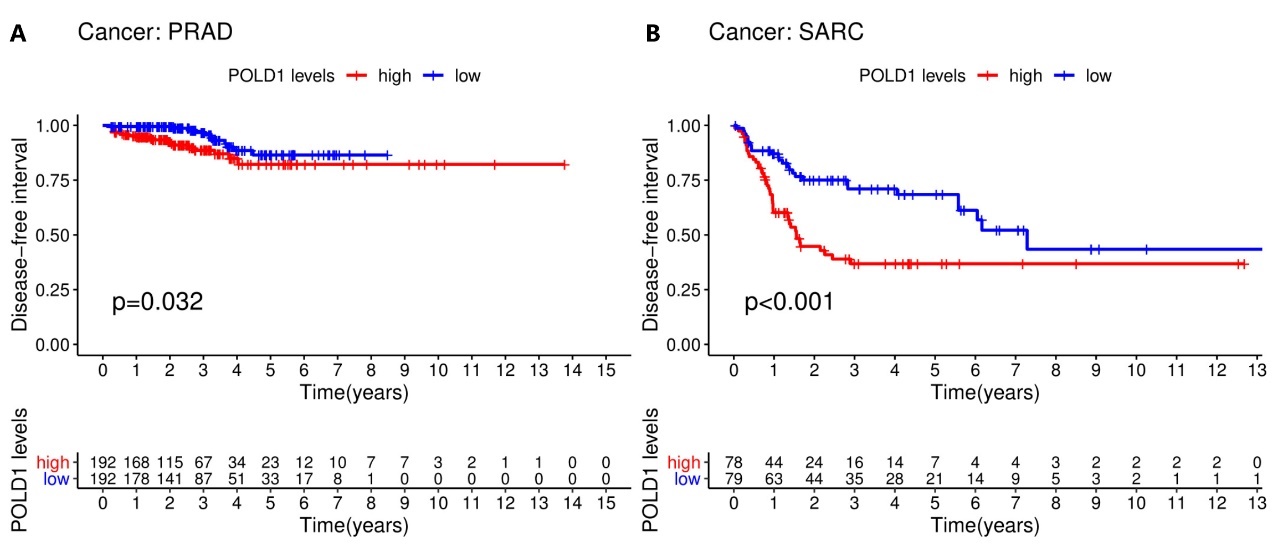

Supplement: Supplementary file 1 — Additional file 1: Figure S1. (A) Comparison of POLD1 mRNA expression in HCC (n = 207) and in normal liver tissues (n = 175) in ICGC-LIRI dataset. Comparison of POLD1 expression in different groups of living status (B), TNM stages (C), and histologic grades (D). (Quasi-likelihood F-test, P< 0.05 was considered significant, * P < 0.05, ** P < 0.01, *** P < 0.001). Figure S2. Kaplan-Meier curves of overall survival for HCC patients with high and low POLD1 mRNA levels from ICGC-LIRI dataset. Figure S3. Effect of POLD1 expression on prognosis and genomic stability in TCGA pan-cancer cohort. Univariate Cox analysis showed the association between POLD1 expression and overall survival (A) or disease-free interval (B). Correlation between POLD1 expression and TMB (C) or MSI (D). Spearman’s correlation coefficients are shown above the bar graphs. (Spearman correlation test, P< 0.05 was considered significant, * P < 0.05, ** P < 0.01, *** P < 0.001). Figure S4. Kaplan-Meier curves and log-rank tests for overall survival of patients with high and low POLD1 mRNA levels in adrenocortical carcinoma (A), diffuse large B-cell lymphoma (B), clear cell renal cell carcinoma (C), kidney renal papillary cell carcinoma (D), brain lower grade glioma (E), HCC (F), lung adenocarcinoma (G), mesothelioma (H), sarcoma (I), and uveal melanoma (J). Figure S5. Kaplan-Meier curves and log-rank tests for disease-free interval of patients with high and low POLD1 mRNA levels in prostate adenocarcinoma (A), and sarcoma (B). [file 12885_2022_9284_MOESM1_ESM.docx]
